# Supplementary material for: Knowledge graph-based recommendation framework identifies drivers of resistance in EGFR mutant non-small cell lung cancer
Source: Nat Commun. 2022 Mar 29;13:1667. doi: 10.1038/s41467-022-29292-7 (PMC8964738; doi:10.1038/s41467-022-29292-7)

Category	Name	Definition	Default
Essentiality	depmap	Gene essentiality according to DepMap; used for filtering only.	exclude
Tractability	trct_ab	Tractability with antibodies	exclude
	trct_sm	Tractability with small molecules	maximize
	trct_om	Tractability with other modalities	exclude
Literature	lit_nsclc	Number of papers mentioning NSCLC AND a candidate gene in cancer context	maximize
	lit_egfr	Number of papers mentioning EGFR AND a candidate gene in cancer context	maximize
	lit_egfr_norm	Normalized frequency EGFR + a gene over total number of papers about a gene	exclude
	lit_nsclc_norm	Normalized frequency NSCLC + a gene over total number of papers about a gene	exclude
KG-derived	L2_egfr	Euclidean distance from a gene to EGFR node based on RESCAL embeddings	exclude
	L2_nsclc	Euclidean distance from a gene to NSCLC node based on RESCAL embeddings	exclude
	n_neighbours	Number of unique neighbours in the full graph	exclude
	n_edges	Number of edges connected to a node in KG	exclude
	degree	Node degree in PPI subgraph	exclude
	pageRank	pageRank for a node computed on PPI subgraph	maximize
	betweenness	betweenness centrality of a node, computed on PPI subgraph	maximize
CRISPR	full_screen	summary consistency across all cell lines in CRISPRn and CRISPRa screens	maximize
	KO_osi	CRISPRn, number of cell lines treated with osimertinib where a gene is a hit	exclude
	KO_gefi	CRISPRn, number of cell lines treated with gefitinib where a gene is a hit	exclude
	KO_all	CRISPRn, number of cell lines treated with either osimertinib or gefitinb where a gene is a hit	exclude
	A_osi	CRISPRa, number of cell lines treated with osimertinib where a gene is a hit	exclude
	A_gefi	CRISPRa, number of cell lines treated with gefitinib where a gene is a hit	exclude
	A_all	CRISPRa, number of cell lines treated with either osimertinib or gefitinib where a gene is a hit	exclude
Clinical	clinical_ES1	mutation enrichment score across internal clinical studies AURAext, AURA2, and AURA3	maximize
	clinical_ES2	mutation enrichment score in internal clinical study FLAURA	maximize
	clinical_ES3	mutation enrichment score comparing internal studies FLAURA and ORCHARD	maximize
Pre-clinical	RNASeq_LFC	log2 fold change from internal RNAseq study, where cancer cell lines were treated with osimertinib vs DMSO treatment	maximize
	RNASeq_pval	adjusted p-values associated with LFC	minimize

Table 1: SUPPLEMENTARY. Definitions of features used for multi-objective optimization. All default features were assigned optimization direction ('maximize/minimize') in the 'Default' column.

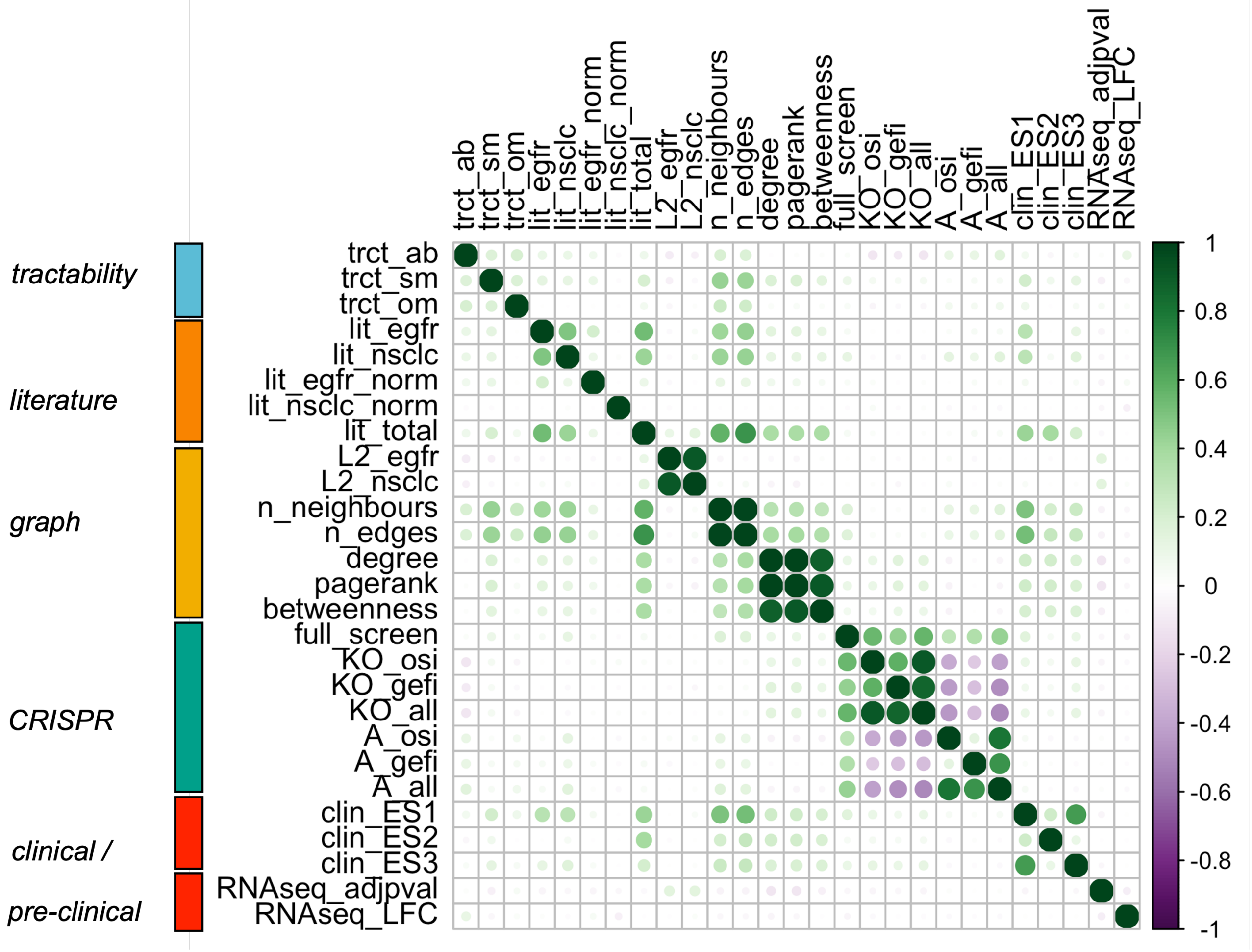

Figure 7: SUPPLEMENTARY. Correlation structure of the hybrid feature space. The analysis indicated two strong underlying patterns: 1) positive correlation within the graph-derived feature group, especially between degree, pageRank and betweenness metrics; 2) summary features derived from the CRISPR screen experiment follow expected pattern with metrics derived from KO and Activation screens demonstrating negative correlation.

**Maps**	**Total**	**p-value**	**FDR**	**In Data**	**Network objects from data**
EGFR signaling in Prostate Cancer	46	7.8e-16	7.9e-13	11	c-Src, PLC-gamma, ERK2 (MAPK1), Tuberin, ERK1 (MAPK3), AKT(PKB), ErbB2, K-RAS, NCOA2 (GRIP1/TIF2), ERK1/2, ErbB3
Development Estrogen-independent activation of ESR1 and ESR2	44	3.1e-14	1.5e-11	10	NCOA1 (SRC1), ERK2 (MAPK1), ERK1 (MAPK3), AKT(PKB), ErbB2, CBP, p300, NCOA2 (GRIP1/TIF2), ERK1/2, ErbB3
Mechanisms of resistance to EGFR inhibitors in lung cancer	45	2.2e-12	7.5e-10	9	c-Src, ERK2 (MAPK1), ERK1 (MAPK3), AKT(PKB), ErbB2, K-RAS, PTEN, HGF receptor (Met), ErbB3
NRF2 regulation of oxidative stress response	54	1.3e-13	2.4e-09	9	ERK2 (MAPK1), BRG1, MafK, ERK1 (MAPK3), AKT(PKB), p53, CBP, KEAP1, PKC
Aberrant B-Raf signaling in melanoma progression	55	1.5e-11	2.4e-09	9	Tuberin, CBP, AKT1, ERK1/2, ROCK, ERK2 (MAPK1), BMF, AKT(PKB), Rictor
Development Androgen receptor in reproductive system development	80	1.7e-11	2.4e-09	10	c-Src, NCOA1 (SRC1), AKT(PKB), p53, CBP, EZH2, AKT1, WT1, NCOA2 (GRIP1/TIF2), ERK1/2
Main pathways of Schwann cells transformation in neurofibromatosis type 1	80	1.7e-11	2.4e-09	10	Neurofibromin, Tuberin, AKT(PKB), Bim, ErbB2, K-RAS, PLC-gamma 1, PTEN, ERK1/2, ErbB3
Androgen receptor activation and downstream signaling in Prostate cancer	110	1.9e-11	2.4e-09	11	c-Src, NCOA1 (SRC1), ERK2 (MAPK1), PKC-delta, ERK1 (MAPK3), AKT(PKB), ErbB2, K-RAS, p53, PTEN, NCOA2 (GRIP1/TIF2)
Regulation of Tissue factor signaling in cancer	43	7.5e-11	8.4e-09	8	ERK2 (MAPK1), ERK1 (MAPK3), AKT(PKB), K-RAS, VEGFR-2, p53, PTEN, ERK1/2
Development Membrane-bound ESR1: interaction with growth factors signaling	45	1.1e-10	1.1e-08	8	c-Src, NCOA1 (SRC1), AKT(PKB), ErbB2, CBP, p300, ERK1/2, ErbB3

Table 2: SUPPLEMENTARY. Pathway Enrichment Analysis. Enrichment analysis consists of matching gene IDs of possible targets with gene IDs in functional ontologies in MetaCore. The probability of a random intersection between a set of IDs the size of target list with ontology entities is estimated using p-value of hypergeometric intersection. The lower p-value means higher relevance of the entity to the dataset, which shows in higher rating for the entity.

Gene	Therapeutic level	Diagnostic level	Prognostic level	Resistance level	FDA level	Oncogenic Alterations (OA)
AKT1	3A	-	-	-	3	-
CDK4	4	-	-	-	3	-
CREBBP	-	Dx2	-	-	-	-
CSF1R	-	-	Px1	-	-	-
ERBB2	1	-	-	-	2	-
EZH2	1	Dx2	Px1	-	2	-
FGFR4	-	-	-	-	-	OA
KDR	-	-	-	-	-	OA
KRAS	1	Dx2	-	R1	2	OA
LZTR1	-	-	-	-	-	OA
MAPK1	-	-	-	-	-	OA
MET	1	-	-	R2	2	-
NFE2	1	-	-	R2	2	likely OA
RICTOR	-	-	-	-	-	OA
SRC	-	-	-	-	-	likely OA
TP53	-	-	Px1	-	-	-
WT1	-	-	Px2	-	-	-
ERBB3	-	-	-	-	-	OA
BCL2L11	-	-	-	-	-	OA
SMARCA4	-	-	-	-	-	OA
MED12	-	-	-	-	-	OA
TSC2	1	-	-	-	2	-
NF1	1	Dx2	-	-	2	-
KEAP1	-	-	-	-	-	OA
PTEN	4	Dx3	-	-	3	
NF2	-	-	-	-	-	likely OA
CIC	-	-	-	-	-	likely OA
CDKN2B	-	-	-	-	-	OA
EP300	-	Dx3	-	-	-	-
GATA6	-	-	-	-	-	likely OA
BCL6	-	Dx2	-	-	-	-

Table 3: SUPPLEMENTARY. Clinical relevance of the recommended genes according to OncoKB [51]. Only genes that can be assigned to OncoKB categories are shown. Specific definitions can be found in OncoKB (`https://www.oncokb.org/levels`).

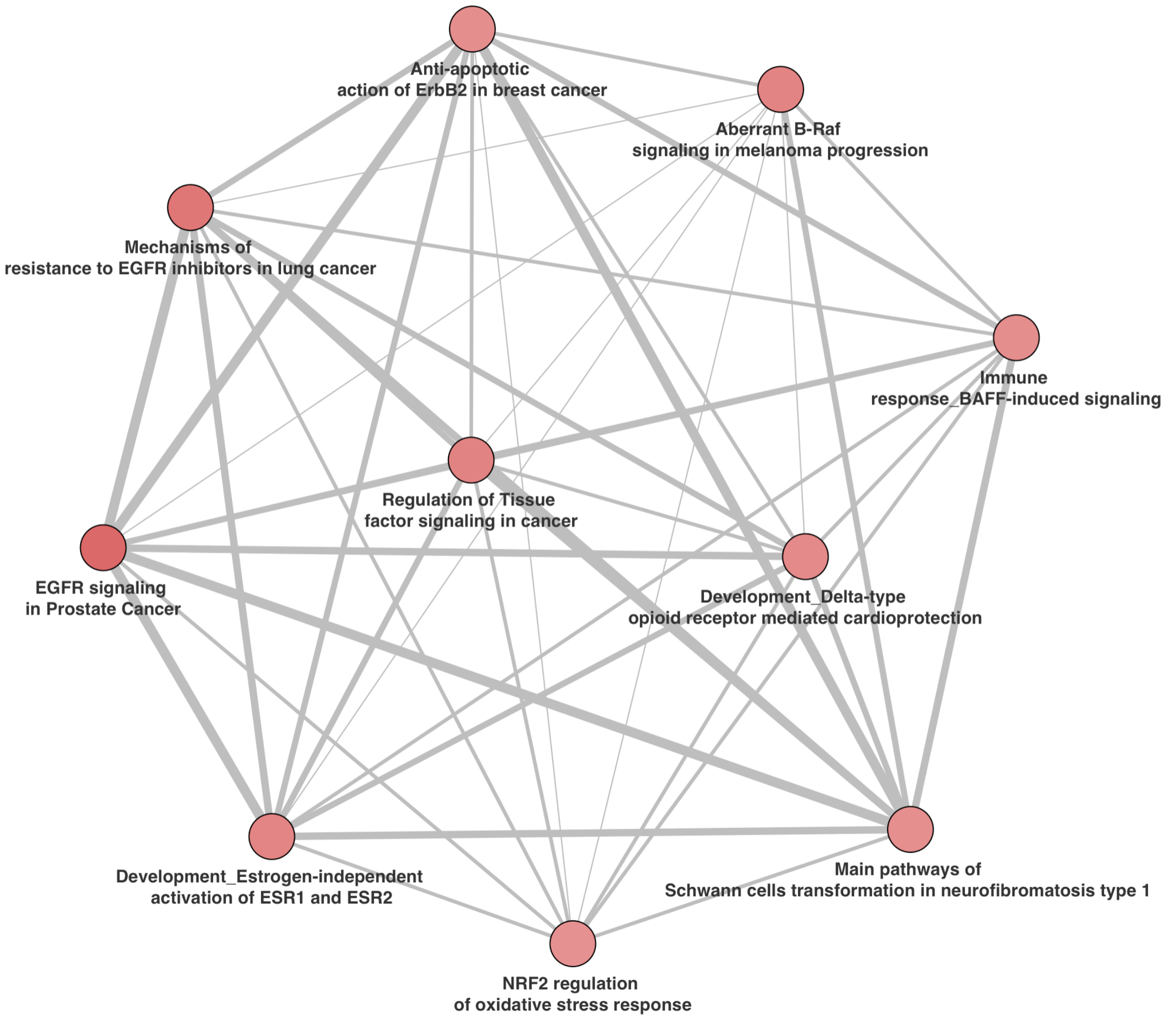

Figure 8: SUPPLEMENTARY. Crosstalk analysis of recommended hits. The network of the top 10 ontology terms from pathway enrichment analysis. Nodes represent top terms and edges represent significant similarities (as measured by hypergeometric test) between these entities. The edge thickness depends on the size of intersection between two ontology terms while the color of the node corresponds to the enrichment z-score.

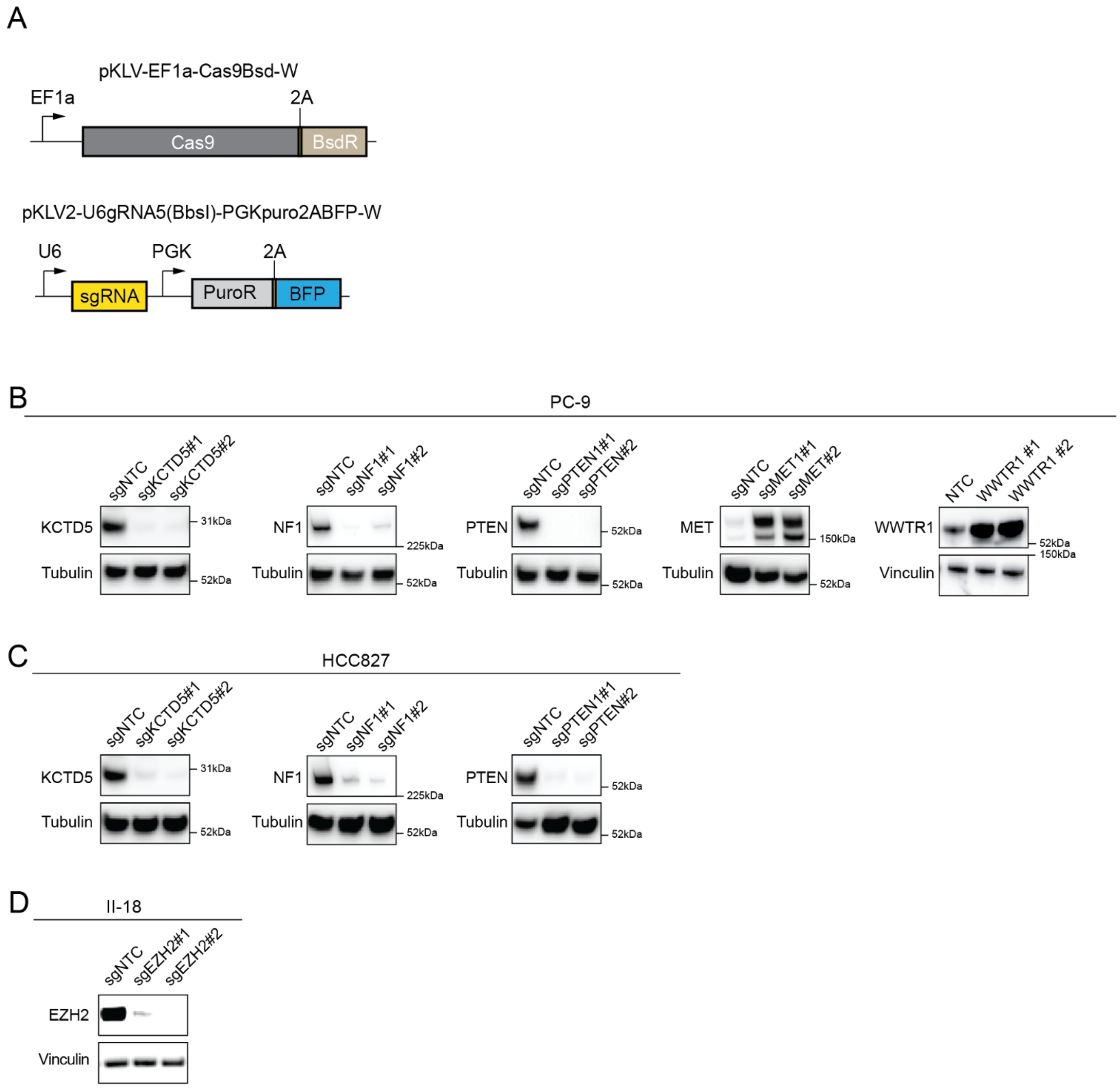

Figure 9: SUPPLEMENTARY. Evaluation of gene KO and activation in NSCLC cell lines. A CRIPSR Cas9 vector system used for generation of PC-9 and HCC827 KO cell lines. In these cell lines Cas9 is expressed constitutively after Blasticidin selection. pKLV2 sgRNA vector co-expressed BFP for FACS based tracking of proliferation. B Loss of target gene expression of KCTD5, NF1 and PTEN as well as overexpression of MET and WWTR1 in PC-9. Whole cell lysates were analysed 14 days after transduction. C Loss of target gene expression of KCTD5, NF1 and PTEN in HCC827. Whole cell lysates were analysed 14 days after transduction. D) Loss of target gene expression of EZH2 in II-18. Whole cell lysates were analysed 14 days after transduction. For each gene KO or activation was confirmed with two independent guide RNAs which are considered biological replicates (n=2).

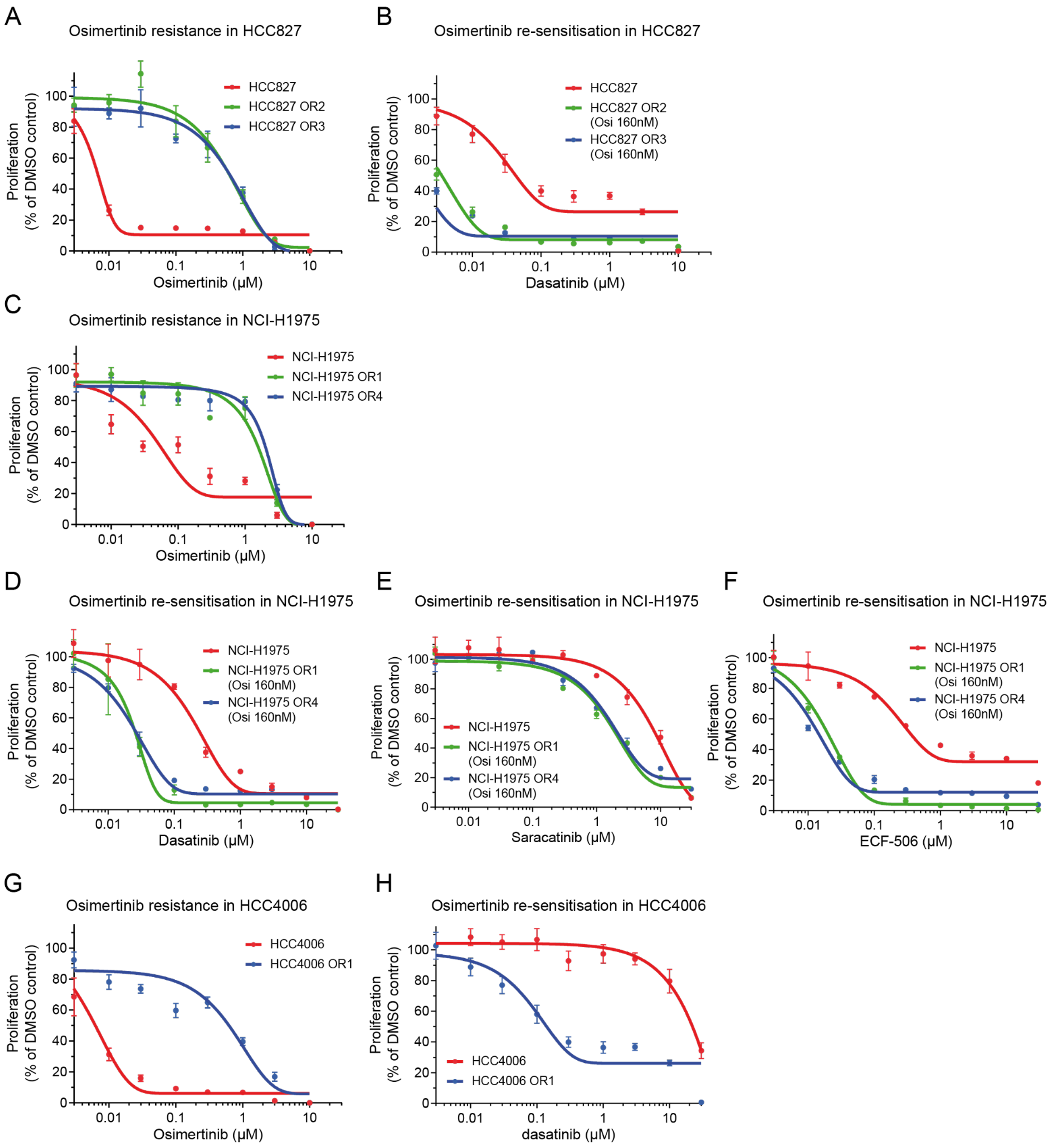

Figure 10: SUPPLEMENTARY. Dose response curves for small molecule SRC inhibitors indicate importance of SRC in mediating osimertinib resistance. A) Osimertinib dose response curve in HCC827 parental cells compared to two lines derived to have acquired osimertinib resistance, as measured by Cell Titer Glo (96h treatment). B) Dasatinib dose response curve in HCC827 parental vs. resistant lines, as measured by Cell Titer Glo (96h treatment). Resistant cells were co-treated with 160 nM osimertinib. C) Osimertinib dose response curve in NCI-H1975 parental cells compared to two lines derived to have acquired osimertinib resistance, as measured by Cell Titer Glo (96h treatment). Dose response curves for NCI-H1975 vs. resistant lines for D) dasatinib E) saracatinib and F) ECF-506, as measured by Cell Titer Glo (96h treatment). G) Osimertinib dose response curve in HCC4006 parental cells compared to two lines derived to have acquired osimertinib resistance, as measured by Cell Titer Glo (96h treatment). H) Dasatinib dose response curve in HCC4006 parental vs. resistant lines, as measured by Cell Titer Glo (96h treatment). Resistant cells were co-treated with 160 nM osimertinib. OR = osimertinib (Osi) resistant. Data are presented as mean values +/- SD (n = 3) of a typical plot, where the experiment was repeated at least 3 times.

**Method**	**Target gene**	**sgRNA**	**Target sequence**
CRISPR KO	NTC	sgNTC	5'-ACGCTAAACCAACGGTGC-3'
	KCTD5	sgKCTD5 #1	5'-ACGTTGAGTCGGACCCACT-3'
		sgKCTD5 #2	5'-GATTTCGGGTCCCGGCACA-3'
	NF1	sgNF1 #1	5'-CCGAAGTTCAGCTGCATGC-3'
		sgNF1 #2	5'-TTAGCAGTTATAAATAGCC-3'
	PTEN	sgPTEN #1	5'-CCGCCAAATTTAATTGCAG-3'
		sgPTEN #2	5'-GATAAGTTCTAGCTGTGGT-3'
	EZH2	sgEZH2 #1	5'-ACTAAGTCTTACCAAATGC-3'
		sgEZH2 #2	5'-AACACCCAACACTTATAAG-3'
CRISPR activation	MET	sgMET #1	5'-TGGCAGGGCAGCGCGCGTGT-3'
		sgMET #2	5'-TGGTCGCCTGGCGGTGCCTC-3'
	WWTR1	sgWWTR1 #1	5'-GTAAAGTACCCATCACGCCC-3'
		sgWWTR1 #2	5'-ACTCAAAGGAATGCAGATGC-3'

Table 4: SUPPLEMENTARY. Target sequences of guide RNAs used in this study

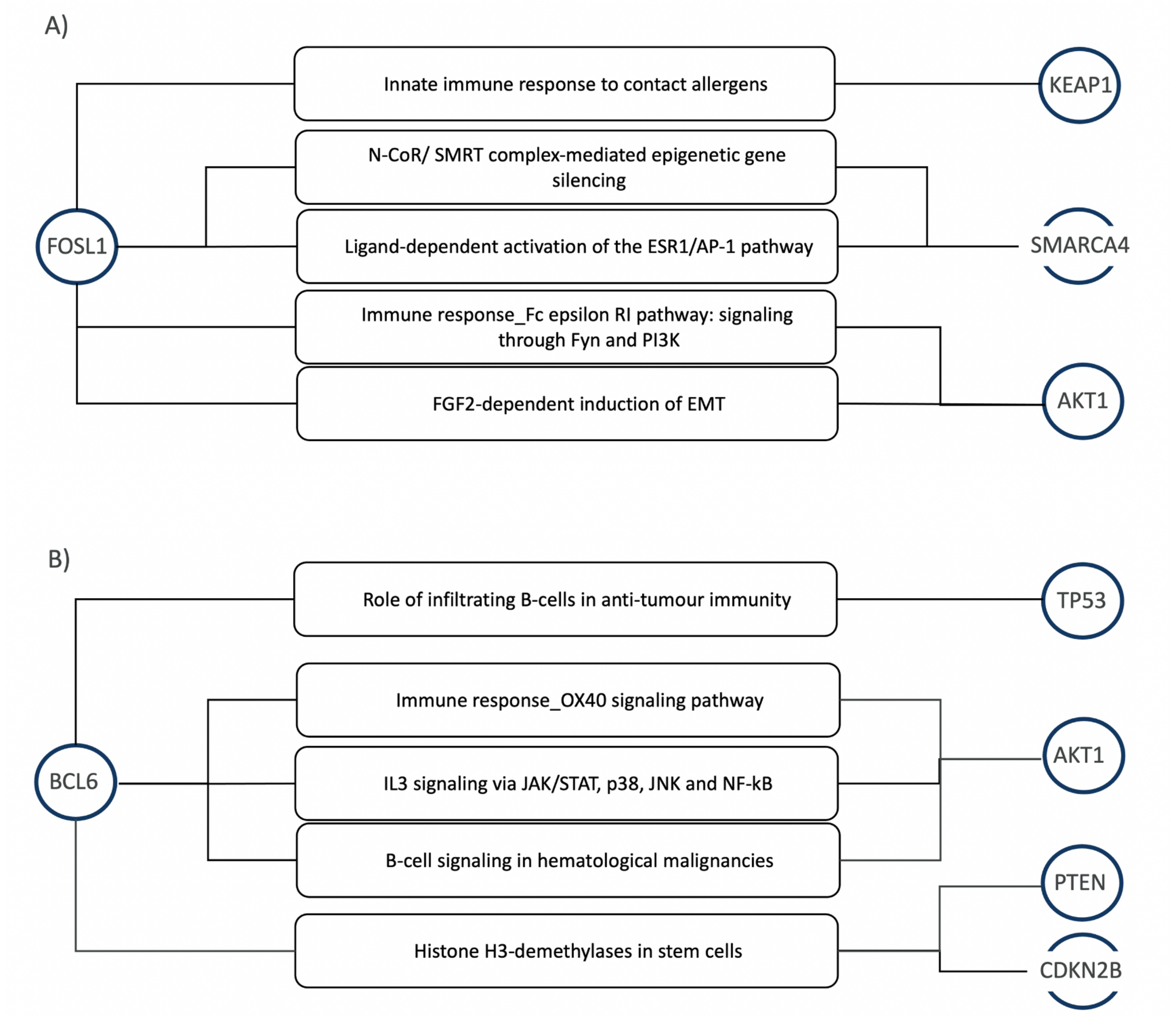

Figure 11: SUPPLEMENTARY. Markers predicted by recommendation system showing evidence paths to osimertinib resistance markers. A) FOSL1's role in epigenetic silencing and innate immune response. B) BCL6's role in anti-tumour immunity, cross-talk with key aberrant pathways in cancer such as JAK/STAT and p38.

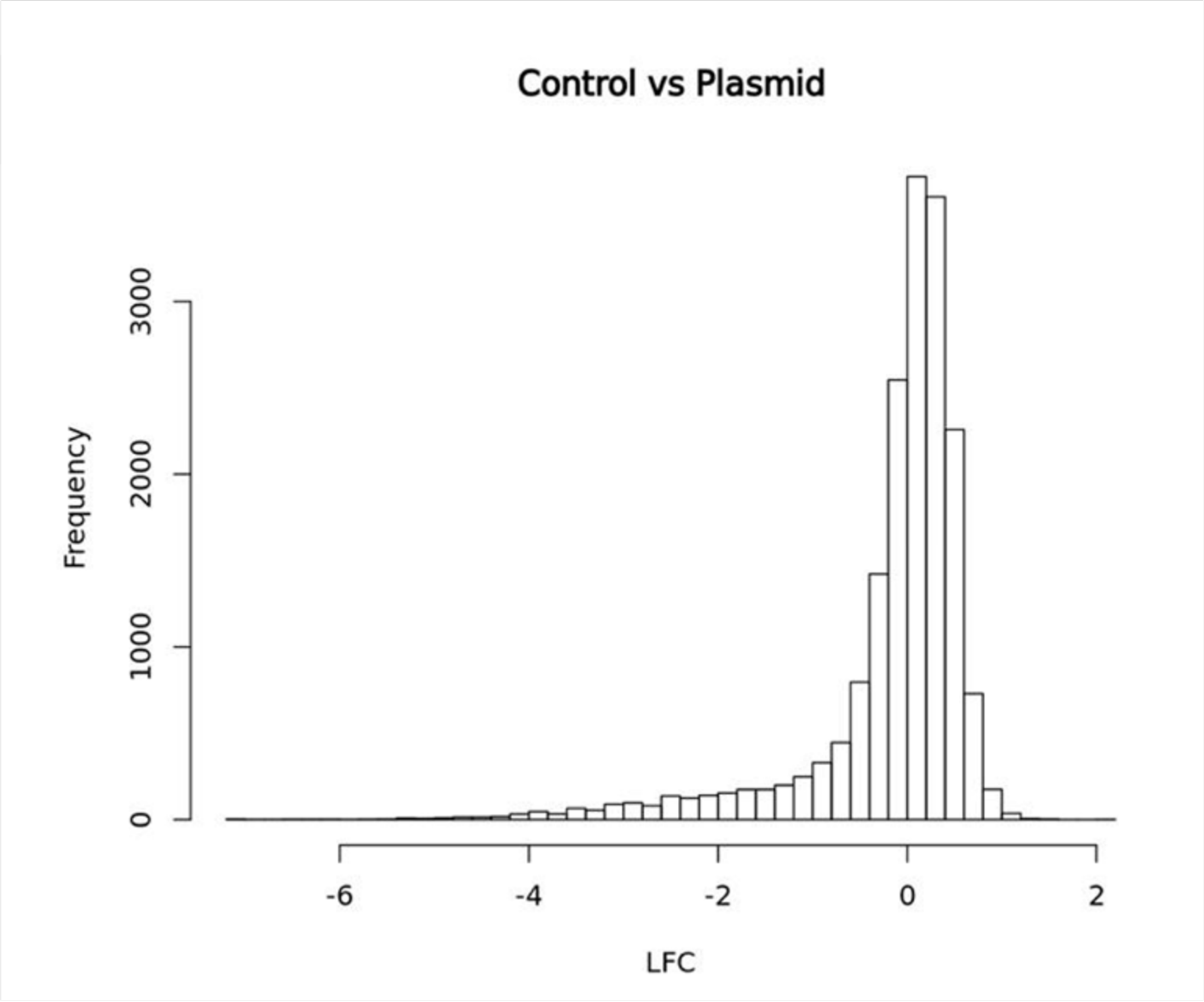

Figure 12: SUPPLEMENTARY. Log Fold Change distribution is expected to be centered around 0 confirming that there are no survival issues with the cell population.

Supplementary Figure 11 Gogleva et al.,

A WB full scans of supplementary figure 9 B/C

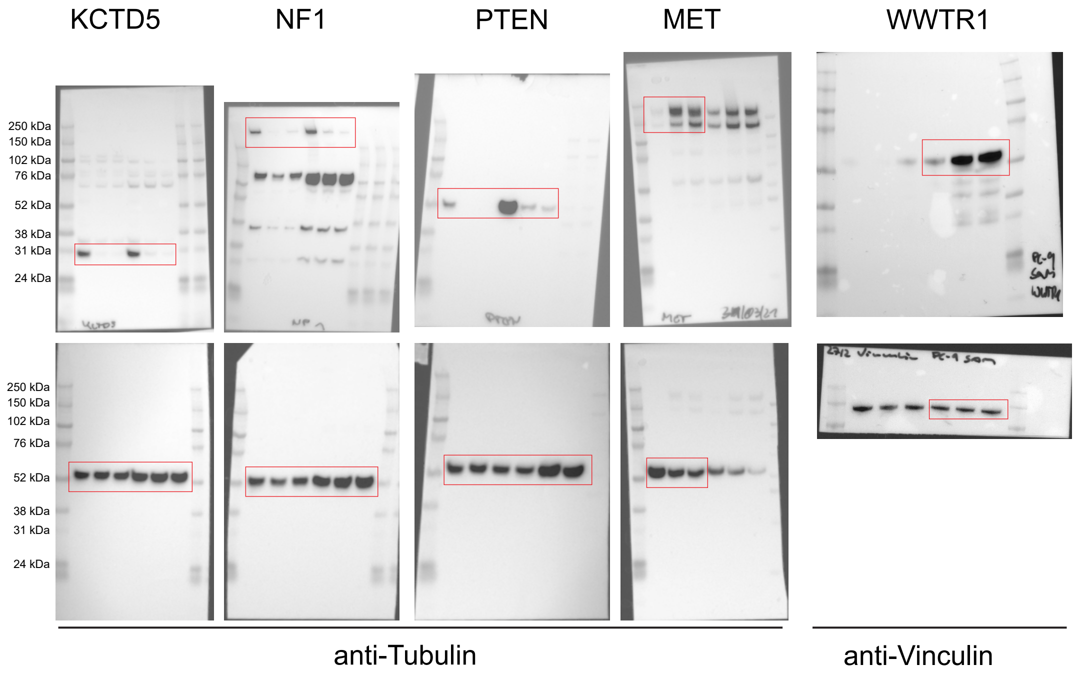

B WB full scans of supplementary figure 9 D

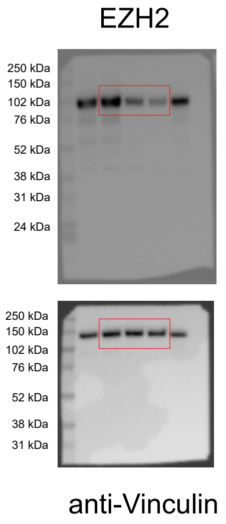

Supplement: Supplementary file 1 — Supplementary Information [file 41467_2022_29292_MOESM1_ESM.pdf]
