# Supplementary material for: Knowledge graph-based recommendation framework identifies drivers of resistance in EGFR mutant non-small cell lung cancer
Source: Nat Commun. 2022 Mar 29;13:1667. doi: 10.1038/s41467-022-29292-7 (PMC8964738; doi:10.1038/s41467-022-29292-7)
Supplement: Supplementary file 2 — Reporting Summary [file 41467_2022_29292_MOESM2_ESM.pdf]

nature portfolio

Corresponding author(s): Krishna C. Bulusu
Eliseo Papa

Last updated by author(s): Feb 25, 2022

Reporting Summary

Nature Portfolio wishes to improve the reproducibility of the work that we publish. This form provides structure for consistency and transparency in reporting. For further information on Nature Portfolio policies, see our Editorial Policies and the Editorial Policy Checklist.

Statistics

For all statistical analyses, confirm that the following items are present in the figure legend, table legend, main text, or Methods section.

n/a	Confirmed	
☐	☒	The exact sample size (n) for each experimental group/condition, given as a discrete number and unit of measurement
☐	☒	A statement on whether measurements were taken from distinct samples or whether the same sample was measured repeatedly
☐	☒	The statistical test(s) used AND whether they are one- or two-sided<br>*Only common tests should be described solely by name; describe more complex techniques in the Methods section.*
☐	☒	A description of all covariates tested
☐	☒	A description of any assumptions or corrections, such as tests of normality and adjustment for multiple comparisons
☐	☒	A full description of the statistical parameters including central tendency (e.g. means) or other basic estimates (e.g. regression coefficient) AND variation (e.g. standard deviation) or associated estimates of uncertainty (e.g. confidence intervals)
☐	☒	For null hypothesis testing, the test statistic (e.g. F, t, r) with confidence intervals, effect sizes, degrees of freedom and P value noted<br>*Give P values as exact values whenever suitable.*
☒	☐	For Bayesian analysis, information on the choice of priors and Markov chain Monte Carlo settings
☐	☒	For hierarchical and complex designs, identification of the appropriate level for tests and full reporting of outcomes
☒	☐	Estimates of effect sizes (e.g. Cohen's d, Pearson's r), indicating how they were calculated

Our web collection on statistics for biologists contains articles on many of the points above.

Software and code

Policy information about availability of computer code

Data collection	A combination of custom and open source code was used to generate features. We used custom python (3.7) and R (4.0.2) scripts to wrangle the data. Graph-derived metrics were computed with neo4j (4.0), RESCAL embeddings were calculated with PyTorch BigGraph library, L2 distances were computed with FAISS (1.7.1). NLP features were extracted with custom Python scripts. User input was collected with Prodigy labelling tool. For more details please see the 'Methods' section and our public code repository (https://github.com/AstraZeneca/skywalkR).<br><br>All libraries and their version numbers are included in this file in our git repository (https://github.com/AstraZeneca/skywalkR/blob/master/renv.lock). The full list is too large to include in the reporting summary.
Data analysis	Custom R (4.0.2) and Python (3.7) scripts were used to analyse the data. For more details please see the 'Methods' section.

For manuscripts utilizing custom algorithms or software that are central to the research but not yet described in published literature, software must be made available to editors and reviewers. We strongly encourage code deposition in a community repository (e.g. GitHub). See the Nature Portfolio guidelines for submitting code & software for further information.

Data

Policy information about availability of data

All manuscripts must include a data availability statement. This statement should provide the following information, where applicable:

- Accession codes, unique identifiers, or web links for publicly available datasets
- A description of any restrictions on data availability
- For clinical datasets or third party data, please ensure that the statement adheres to our policy

All data generated, analysed, and interpreted have been included as part of the code repository (https://github.com/AstraZeneca/skywalkR). Specifics have been

included in the Methods section. For clinical data features, aggregated enrichment scores have been included, but not individual patient-level data as these are proprietary at the time of drafting the manuscript. However, these trials have been published and appropriately referenced in the manuscript. Two RNAseq features we use in our study, which is currently unpublished but has been uploaded to Gene Expression Omnibus with an accession ID: GSE193259. As you will appreciate, this is currently under embargo till the publication describing the study is released. The two features however have been included in our Git repository for your readers to access already with a brief description of the RNAseq study in our Methods section. Data Availability statement from the manuscript below:

All data generated, analysed, and interpreted have been included as part of the code repository https://github.com/AstraZeneca/skywalkR[47]. Specifics have been included in the Methods section. For clinical data features, aggregated enrichment scores have been included, but not individual patient-level data as these are proprietary at the time of drafting the manuscript. However, these trials have been published and appropriately referenced in the manuscript. We utilised two features from an internal RNAseq study that we described briefly in the additional methods document. The data itself has been included in our code repository for reviewers and readers to access.The raw RNAseq data used in this study will be accessible in the GEO database under accession code GSE193259 https://www.ncbi.nlm.nih.gov/geo/query/acc.cgi?acc=GSE193259. The Knowledge Graph as it was used for this study cannot be released due to proprietary data included in it. However, the construction of the KG was as described in Geleta et al.[33]

Field-specific reporting

Please select the one below that is the best fit for your research. If you are not sure, read the appropriate sections before making your selection.

☒ Life sciences ☐ Behavioural & social sciences ☐ Ecological, evolutionary & environmental sciences

For a reference copy of the document with all sections, see nature.com/documents/nr-reporting-summary-flat.pdf

Life sciences study design

All studies must disclose on these points even when the disclosure is negative.

Sample size	Cell line sample sizes were chosen based on the number of replicates required to reach a statistically confident conclusion of the observation, also mentioned in figure captions. No in vivo or clinical study was performed as part of this study.
Data exclusions	No data were excluded from the study. Wherever patient data was utilised, an aggregated version was utlised in accordance with clinical data policy to not disclose individual patient-level information. All clinical studies used have been published and appropriately referenced in the manuscript.
Replication	Data analysis results can be replicated by implementing the pipeline using the same default settings as described in our code repository (https://github.com/AstraZeneca/skywalkR).
Randomization	Not applicable to our study as we do not utilise raw readouts from studies that requires randomization. The preclinical experimental validation we performed did not require randomisation. No in vivo or clinical study was performed as part of this study.
Blinding	Not applicable to our study as we do not utilise raw readouts from studies that requires blinding. The preclinical experimental validation we performed did not require blinding. No in vivo or clinical study was performed as part of this study.

Reporting for specific materials, systems and methods

We require information from authors about some types of materials, experimental systems and methods used in many studies. Here, indicate whether each material, system or method listed is relevant to your study. If you are not sure if a list item applies to your research, read the appropriate section before selecting a response.

Materials & experimental systems

n/a	Involved in the study	
☐	☒	Antibodies
☐	☒	Eukaryotic cell lines
☒	☐	Palaeontology and archaeology
☒	☐	Animals and other organisms
☒	☐	Human research participants
☐	☒	Clinical data
☒	☐	Dual use research of concern

Methods

n/a	Involved in the study	
☒	☐	ChIP-seq
☒	☐	Flow cytometry
☒	☐	MRI-based neuroimaging

Antibodies

Antibodies used	*Describe all antibodies used in the study; as applicable, provide supplier name, catalog number, clone name, and lot number.*
Validation	*Describe the validation of each primary antibody for the species and application, noting any validation statements on the manufacturer's website, relevant citations, antibody profiles in online databases, or data provided in the manuscript.*

Eukaryotic cell lines

Policy information about cell lines

Cell line source(s)	PC-9 was purchased from ECACC and NCI-H1975, HCC4006 and HCC827 cell lines were purchased from ECACC and ATCC respectively. IL-18 cell line was obtained from Tohoku University (TKG0177).
Authentication	All cell lines used in these studies were authenticated using STR profiling (IDEXX Laboratories).
Mycoplasma contamination	Yes – confirmed mycoplasma negative by AZ cell bank for validation studies.. Mycoplasma testing was carried out using the MycoSEQ Mycoplasma detection kit (ThermoFisher).
Commonly misidentified lines (See ICLAC register)	PC9 used to be called PC-14.

Clinical data

Policy information about clinical studies

All manuscripts should comply with the ICMJEguidelines for publication of clinical research and a completedCONSORT checklist must be included with all submissions.

Clinical trial registration	We utilised data from the following published studies, and not generated data for the purpose of our manuscript: NCT02151981, NCT02296125, NCT01802632, NCT02094261, NCT03944772.
Study protocol	The full trial protocols are available in the respective publications/references, cited in the main manuscript. Accessible via respective publications referenced in manuscript
Data collection	Accessible via respective publications referenced in manuscript. No clinical study was performed as part of this study.
Outcomes	Accessible via respective publications referenced in manuscript. No clinical study was performed as part of this study.
